# 17p11.2 and Xq28 duplication detected in a girl diagnosed with Potocki–Lupski syndrome

**DOI:** 10.1186/s13104-015-1439-7

**Published:** 2015-09-29

**Authors:** Dulika S. Sumathipala, Eranda N. Mandawala, Samanmalee P. Sumanasena, Vajira H. W. Dissanayake

**Affiliations:** Human Genetics Unit, Faculty of Medicine, University of Colombo, Colombo, Sri Lanka; Asiri Center for Genomic and Regenerative Medicine, Narahenpita, Sri Lanka; Faculty of Medicine, University of Kelaniya, Kelaniya, Sri Lanka

**Keywords:** Potocki–Lupski, Multiplex ligation probe amplification, Sri Lanka

## Abstract

**Background:**

Potocki–Lupski syndrome is a microduplication syndrome associated with duplication at 17p11.2. Features include facial dysmorphism, moderate to mild cognitive impairment and behavioural abnormalities including autism spectrum disorders.

**Case presentation:**

We describe a patient from Sri Lanka that was referred for genetic assessment at 4 years of age due to subtle facial dysmorphism and expressive language impairment. She was diagnosed with Potocki–Lupski syndrome through multiplex ligation probe amplification. She carried two duplications; one in 17p11.2 consistent with Potocki–Lupski, and one in Xq including the region for X-linked intellectual disability.

**Conclusion:**

Despite the absence of expected behavioural symptoms, many features of this patient are in accordance with Potocki–Lupski syndrome. This is the first diagnosed patient in Sri Lanka.

## Background

Potocki–Lupski syndrome (PTLS) is associated with a microduplication of chromosome 17p11.2 [1]. The clinical features of PTLS typically include infantile hypotonia, poor feeding, failure to thrive, developmental delay, intellectual disability, speech and language impairment, behavioural abnormalities and autistic spectrum disorder [2]. The phenotypic spectrum in adults with PTLS has not been determined.

The genetic mechanism underlying PTLS has been recognised as Non Allelic Homologous Recombination (NAHR) at the 17p11.2 chromosomal region [3]. This region is known to be rich in low copy repeats which is a major cause for DNA rearrangements and associated genomic disorders.

Genetic diagnosis of PTLS has been based on chromosome banding, fluorescent in situ hybridisation, multiplex ligation probe amplification and recently genomewide microarray. This is the first case report describing the diagnosis of PTLS through the use of multiplex ligation probe amplification (MLPA) in Sri Lanka.

## Case presentation

We report a 4 year old female with PTLS (OMIM 610883) from Sri Lanka who was diagnosed with duplication 17p11.2 by multiplex ligation probe amplification performed due to severe expressive speech impairment. She was a product of non-consanguineous parents, born at 37 weeks gestation by lower segment caesarean section due to maternal gestational diabetes, with a birth weight of 2.885 kg. There was no history of infantile hypotonia. Developmental milestones were delayed with walking achieved at 2 years of age. Speech delay was noted and at presentation she was capable of speaking a maximum of two words together with inability to construct sentences. Receptive language skills were not impaired. Hearing showed no clinically apparent deficit. Vision was impaired with hypermetropia, and she used spectacles. She was cheerful and engaged well socially. Common symptoms of autism including avoidance of eye contact, repetitive behaviour, fixations and lack of interest in social interaction were not present. She had borderline to mild intellectual disability based on clinical evaluation and was schooling with peers. There was no history of cardiac defects or sleeping problems. On examination she had distinctive facial features including triangular face, wide forehead, slightly downslanting palpebral fissures, prominent tip of nose, smooth philtrum, and dental malocclusion. Hands showed clinodactyly of the 5th finger bilaterally and an increased gap between the first and second toe of the right foot.

## Methods

**Fig. 1:**
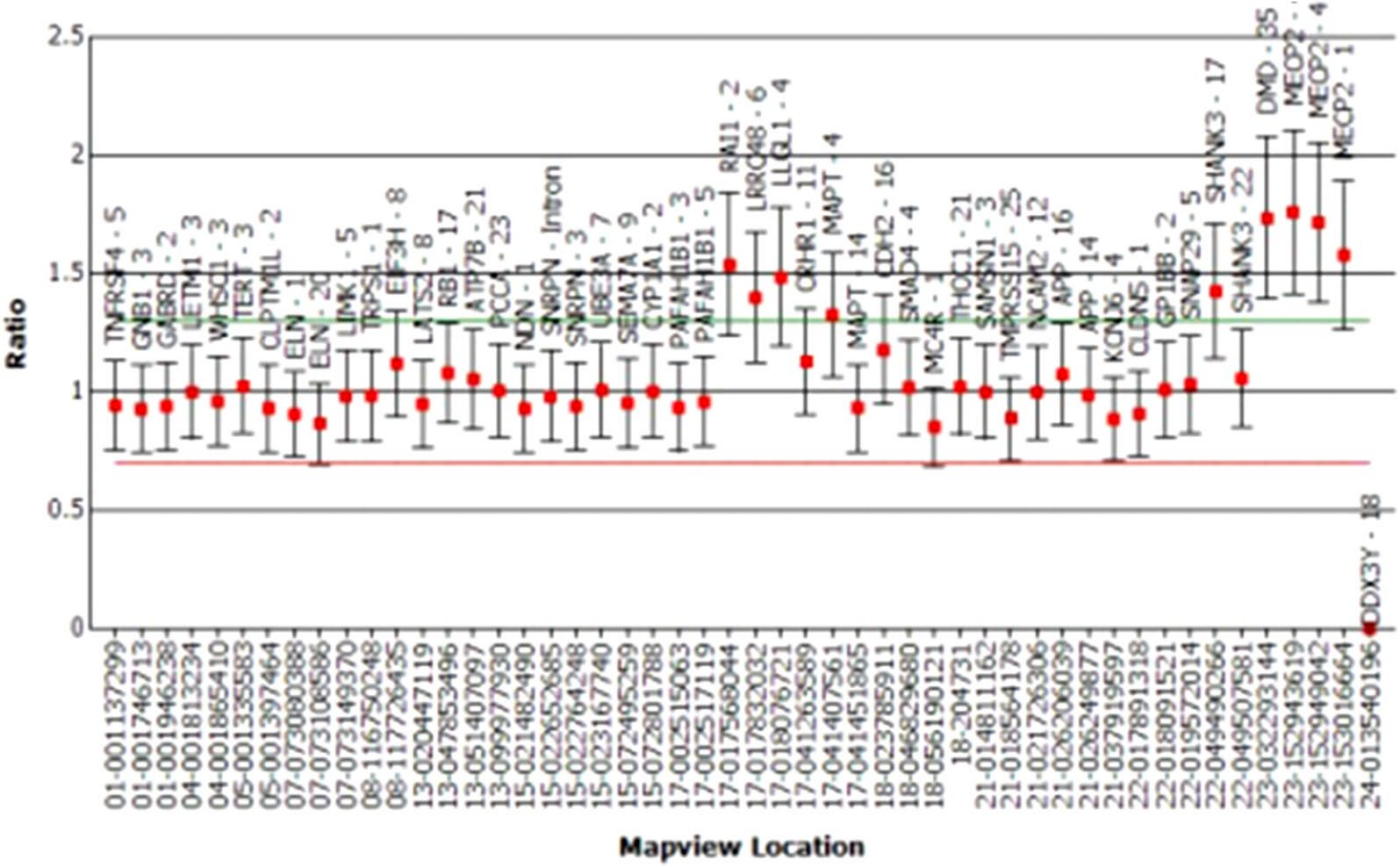
Multiplex ligation probe amplification output showing duplication at 17p11.2 and Xq28 regions

Genetic analysis was performed using multiplex ligation probe amplification (MLPA). The SALSA^®^ MLPA^®^ P245 Microdeletion Syndromes-1 probemix (MRC Holland, Amsterdam, The Netherlands) that has been developed to screen patients presenting with unexplained developmental delay and/or mental retardation for multiple microdeletion syndromes was used. This revealed a duplication of 17p11.2. The region included the *retinoic acid inducible 1* gene *(RAI1)* gene which is compatible with PTLS. Apart from the *RAI1* gene *leucine rich repeat containing 48* (*LRRC48)* and *lethal giant larvae homolog 1 (LLGL1)* genes in the 17p11.2 chromosomal region were duplicated. In addition the Xq28 chromosomal region which includes *methyl CpG binding protein 2 (MECP2)* gene showed duplication (Fig. 1).

All procedures adhered to institutional guidelines (Ethics Review Committee, Faculty of Medicine, University of Colombo) and to the Helsinki Declaration.

## Discussion

This is the first report of PTLS diagnosis in Sri Lanka. Clinically the patient presented with speech impairment and mild facial dysmorphism. However unlike the majority of previously reported cases of PTLS behavioural symptoms including autism spectrum disorders were not present. Genetic diagnosis of PTLS was made using MLPA, which showed 17p11.2 duplication. In addition she also carried duplication in the Xq28 chromosomal region.

Genetic disorder assessment previously limited to microscopic chromosomal anomalies and single nucleotide polymorphisms (SNP) detected by traditional polymerase chain reaction (PCR) based sequencing has expanded following the detection of copy number variations (CNV). Many sporadic microdeletions, microduplication syndromes have been recognised to occur due to CNV. Disease occurrence is either due to altered copy number of genes susceptible to dosage effect or changes in the regulatory sequences. Detection of such CNV requires locus specific testing either through fluorescent in situ hybridisation, multiplex ligation probe amplification or high resolution screening of the entire genome through microarray technology. In resource limited settings such as Sri Lanka, MLPA techniques provide an opportunity to arrive at a genetic diagnosis. However, genome wide screening through microarray technology should be the next step in diagnostics.

The low copy repeats found in the proximal short arm of chromosome 17 region has been identified as a major cause of DNA rearrangements associated with many genomic disorders [1]. PTLS which is due to duplication 17p11.2, is associated with duplication of the *retinoic acid inducible 1* gene (*RAI1*), which was seen in our patient [4]. The MLPA mix used in the microduplication, microdeletion testing included probes for 22 syndromes, including Smith Magenis syndrome (SMS). As the reciprocal microduplication syndrome of SMS, detection of PTLS was possible through *RAI1* gene dosage. The phenotype of this patient was relatively mild and had the possibility of being unrecognized despite the varied medical symptoms. Therefore referral for genetic evaluation in the presence of delay in global or selected milestones is useful to obtain an etiological diagnosis despite the limitations in sensitivity and specificity of methods used at present.

*MECP2* duplication syndrome is a condition that occurs almost exclusively in males and manifest as developmental delay, infantile hypotonia, absent speech and lack of ambulation [5]. This is thought to be due to skewed X chromosome inactivation with the resulting inactivation of the duplication containing X chromosome in females. Therefore despite similar clinical features in PTLS and *MECP2* duplication syndromes, the clinical features noted in the patient were assumed to be associated with PTLS syndrome. However recently females with *MECP2* duplication have been found to manifest neurobehavioral and psychiatric symptoms [6]. Therefore further investigation is ideal to confirm skewed X chromosome inactivation. The patient lacked significant behavioural or psychiatric symptoms which are of additional significance to discount the clinical effect possibly caused by duplication of *MECP2*. However the dual duplication of the two chromosomal regions of the genome in this patient was interesting to note.

## Conclusion

This case report describes the clinical features and genetic findings of a 4 year old girl diagnosed with Potocki–Lupski syndrome. Symptoms and signs are in accordance with reported clinical features of PTLS. Genetic diagnosis through MLPA has revealed duplications in 17p11.2 and Xq28. This includes the critical *RAI1* gene which has been shown to be associated with PTLS.

## Consent

Written informed consent was obtained from the patients’ parents for publication of this Case report and any accompanying images. A copy of the written consent is available for review by the Editor of this journal.

## Abbreviations

PTLS: Potocki–Lupski syndrome
NAHR: non allelic homologous recombination
MLPA: multiplex ligation probe amplification
*RAI1*: *retinoic acid inducible 1* gene
*LRRC48*: *leucine rich repeat containing 48* gene
*LLGL1*: *lethal giant larvae homolog 1* gene
*MECP2*: methyl CpG binding protein 2 gene
SNP: single nucleotide polymorphisms
PCR: polymerase chain reaction
CNV: copy number variations
SMS: Smith Magenis syndrome
